# Image-guided transplantation of single cells in the bone marrow of live animals

**DOI:** 10.1038/s41598-017-02896-6

**Published:** 2017-06-20

**Authors:** Raphaël Turcotte, Clemens Alt, Judith M. Runnels, Kyoko Ito, Juwell W. Wu, Walid Zaher, Luke J. Mortensen, Lev Silberstein, Daniel C. Côté, Andrew L. Kung, Keisuke Ito, Charles P. Lin

**Affiliations:** 1 Advanced Microscopy Program, Center for Systems Biology and Wellman Center for Photomedicine, Massachusetts General Hospital, Harvard Medical School, Boston, MA 02114 USA; 2 Department of Biomedical Engineering, Boston University, Boston, MA 02215 USA; 3 Ruth L/ and David S. Gottesman Institute for Stem Cell, Regenerative Medicine Research, Department of Cell Biology and Stem Cell Institute, Albert Einstein College of Medicine, Bronx, NY 10461 USA; 4 Stem Cell Unit, Department of Anatomy, College of Medicine, King Saud University, Riyadh, 11461 Saudi Arabia; 5 Regenerative Bioscience Center, Rhodes Center for ADS, and College of Engineering, University of Georgia, Athens, GA 30602 USA; 6 Center for Regenerative Medicine, Massachusetts General Hospital, Harvard Medical School, Boston, Massachusetts 02114 USA; 7 Département de Physique, Génie Physique et Optique and Centre de recherche de l’Institut Universitaire en Santé Mentale de Québec, Université Laval, Québec City, Québec G1J 2G3 Canada; 8 Memorial Sloan Kettering Cancer Center, New York, NY 10065 USA; 9 Department of Medicine, Albert Einstein Cancer Center, Albert Einstein College of Medicine, Bronx, NY 10461 USA; 10 Harvard Stem Cell Institute, Cambridge, MA 02138 USA

## Abstract

Transplantation of a single hematopoietic stem cell is an important method for its functional characterization, but the standard transplantation protocol relies on cell homing to the bone marrow after intravenous injection. Here, we present a method to transplant single cells directly into the bone marrow of live mice. We developed an optical platform that integrates a multiphoton microscope with a laser ablation unit for microsurgery and an optical tweezer for cell micromanipulation. These tools allow image-guided single cell transplantation with high spatial control. The platform was used to deliver single hematopoietic stem cells. The engraftment of transplants was tracked over time, illustrating that the technique can be useful for studying both normal and malignant stem cells *in vivo*.

## Introduction

The bone marrow (BM) contains a rare population of hematopoietic stem cells (HSCs) from which all blood cells are derived. The BM also contains a population of stromal cells, or mesenchymal stem cells (MSCs), that give rise to skeletal and connective tissues. In addition, blood cancers such as leukemia can emerge from malignant transformation of hematopoietic stem and progenitor cells (HSPCs) within the BM, and many solid tumors can seed the BM with disseminated tumor cells or cancer stem cells that can develop into bone metastasis. Understanding how the normal and malignant stem cells interact with the BM microenvironment is therefore of fundamental and clinical importance^1, 2^.

The stem cell niche is a specialized microenvironment where the stem cells reside and receive critical extrinsic signals that regulate their quiescence, self-renewal, and differentiation. Despite intense efforts, detailed characterization of the HSC niche has been enormously challenging due to the complexity of the BM microenvironment, its location deep inside the bone matrix, and lack of specific markers illuminating the various stem cell components. The organization of the BM MSC and CSC niches are even less well understood. One useful experimental approach has been HSC transplantation because of its clinical significance and because sophisticated techniques are available for harvesting donor HSCs and analyzing recipient hematopoietic reconstitution post-transplantation. Donor stem cells can be specifically tagged and tracked using an exogenous label, enabling direct visualization by intravital microscopy of the location of HSC homing and engraftment with single cell resolution^3, 4^. However, it is not possible to directly assess the function of the individual cells being imaged, since the outcome of the transplantation could only be assessed at the population level.

On the other hand, a single, intravenously injected HSC has the remarkable functional capacity to rescue a lethally-irradiated mouse and reconstitute the entire hematopoietic system long-term provided that it is co-transplanted with the appropriate radio-protective hematopoietic cell population for short-term support^5–8^. However, the single injected HSC cannot be imaged; only its progeny can be visualized after the stem cell has undergone extensive proliferation^9^, leaving open the question of the initial site of HSC engraftment and how the HSC interacts with its niche. Thus there is need for a technique that can track an individual stem cell from its initial engraftment to its long-term regenerative potential. Here we describe an optical platform that enables controlled transplantation of single cells directly into a defined location in the calvarial bone marrow (BM) of live mice. The same delivery site can be revisited in subsequent imaging sessions enabling the assessment of the transplanted cell engraftment.

## Results

### Approach to single cell transplantation

**Figure 1:**
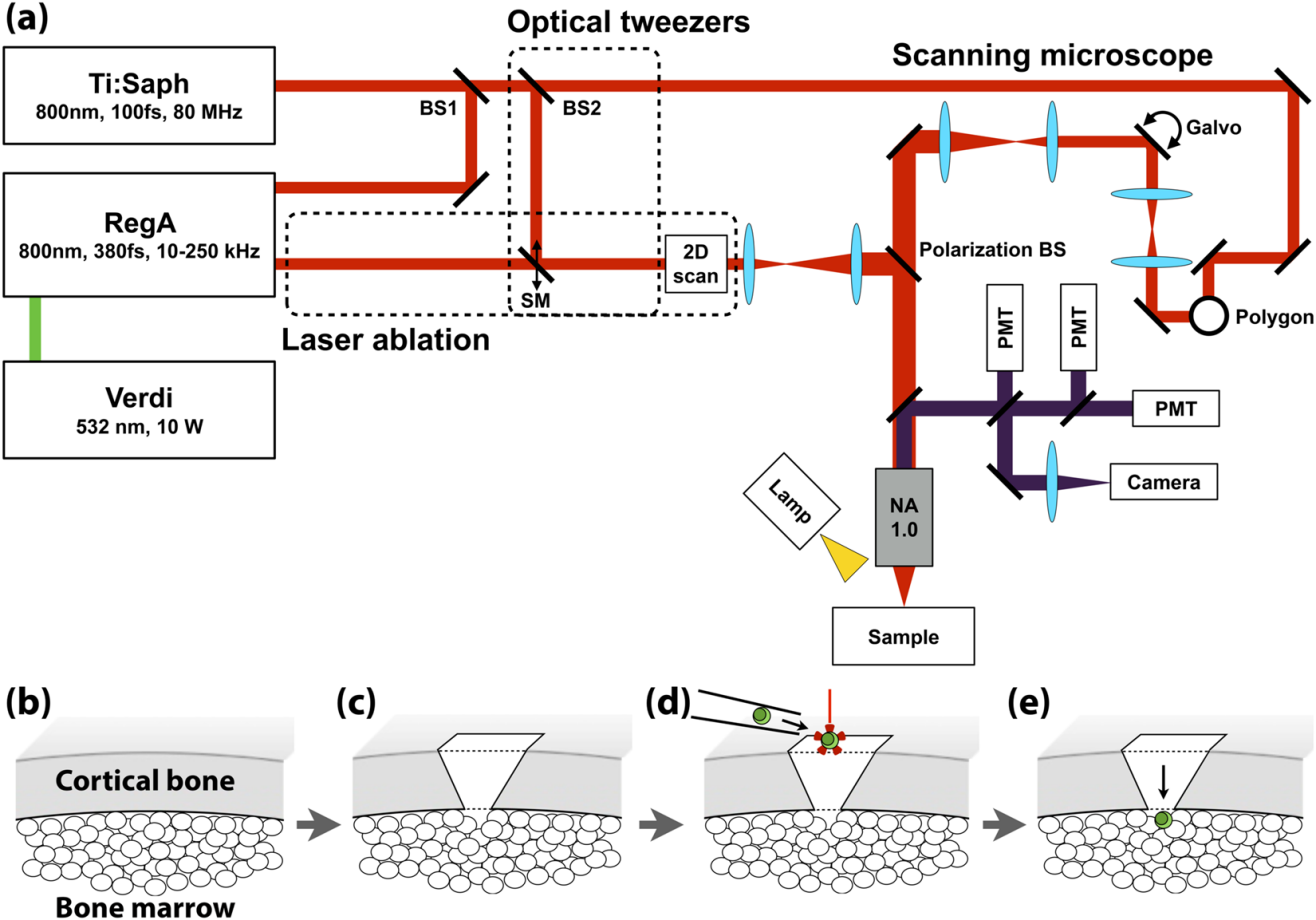
The optical platform for image-guided single cell transplantation. (**a**) Schematic of the optical system that integrates a video-rate laser scanning multiphoton microscope with a laser ablation beam for microsurgery and an optical tweezer for single cell trapping. (**b**) Second harmonic imaging is used to identify a location for transplantation. (**c**) The ablation laser beam is used to cut a delivery channel through the cortical bone, leaving only a hole slightly larger than the size of a cell connecting to the bone marrow cavity. (**d**) A single cell is released from a glass micropipette into the optical tweezer above the channel and (**e**) then directed by the optical trap through the opening.

The platform consists of a video-rate multiphoton laser scanning microscope^10^, a femtosecond laser ablation unit^11^, and a laser tweezer (Fig. 1a, see Methods). We performed single cell transplantation following a four-step approach (Fig. 1b–e). First, the delivery site is selected by 3D video-rate scanning of the calvarium to locate an appropriate BM cavity that is reachable within 100 µm beneath the bone surface (Supplementary Fig. 1 and Supplementary Movie 1). Second, femtosecond laser ablation is used to create a microchannel in the bone. The cell is then brought on top of the microchannel in a glass micropipette and transferred to the laser tweezer. Finally, the optically trapped cell is passaged through the microchannel to the BM under the control of the laser tweezer.

**Figure 2:**
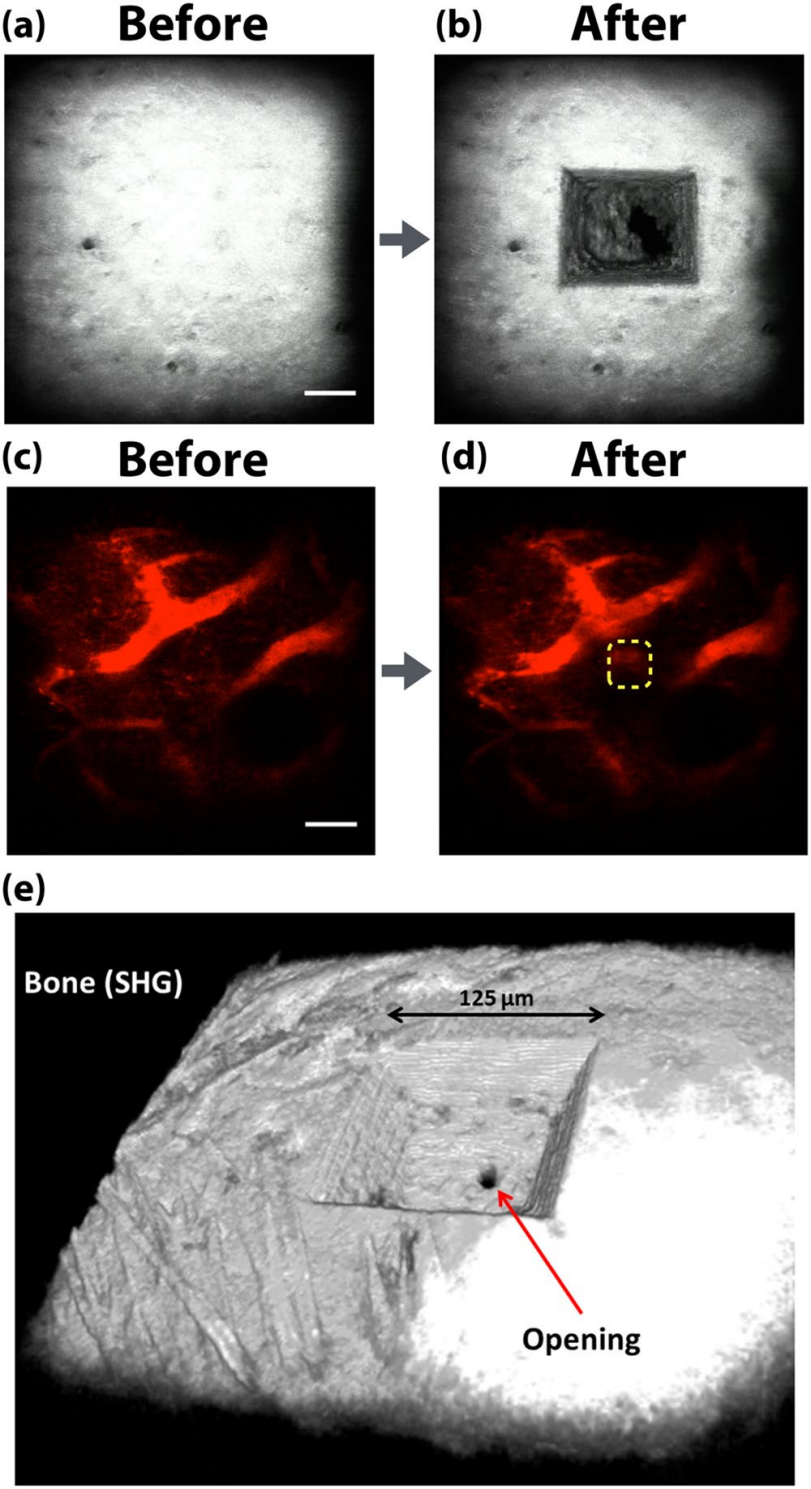
Laser microsurgical bone removal and evaluation of tissue integrity. Images (**a–d**) are maximum intensity projections. Cortical bone images taken before (**a**) and after (**b**) ablation, showing the microchannel channel with the opening to the BM at the bottom. (**e**) Is a 3D reconstructed image of the ablated microchannel. The BM being highly vascularized, the delivery sites are selected to minimize damage to the vasculature during femtosecond laser ablation. Vasculature images taken before (**c**) and after (**d**) ablation, showing the intact blood vessels adjacent to the ablation site. White: SHG, Red: Rhodamine-6G,70kD dextran. Scale bars 50 μm.

All operations are performed under image guidance, enabling single cells to be deposited at specific locations in the BM with minimal perturbation to surrounding tissues. A single femtosecond laser source at 800 nm is used to simultaneously image the bone, the cells of interest and the vascular network (Methods). The bone is visualized using the intrinsic collagen second harmonic generation (SHG) signal (Fig. 2a). Knowing the vascular architecture is important in order to avoid disrupting blood vessels in the bone removal step. The vasculature is therefore simultaneously imaged by two-photon-excited fluorescence (TPEF) of an intravenously infused fluorescent dye (Fig. 2c). Additional landmarks such as specific niche cell populations expressing a fluorescent protein distinct from the donor cell fluorescent protein can also be used to help define the delivery site.

### Laser microsurgical bone removal

In order to gain access to the BM, a microchannel is created through the hard bone matrix using femtosecond laser ablation (Fig. 2b and Supplementary Movie 2). An ideal delivery channel is one that is slightly larger than the size of the cell to be delivered. A portion of the same 800 nm laser source is used to seed a regenerative amplifier, which produces pulses with the energy needed for ablation. A single 225 nJ pulse (radiant exposure of ~7.5 J/cm^2^) removes about ~4 µm^3^ of bone at the focus of a 60X 1.0 NA water immersion objective lens, with minimal collateral damage to surrounding tissue^11–14^. Use of a high numerical aperture objective requires that a V-shaped channel be created with a larger opening at the top, tapering down to an opening of less than 2 cell diameters at the entrance to the BM cavity (Fig. 2e). Channel shape is controlled by moving the laser focus with a pair of galvanometric scanners and a computer-controlled stage, while chiseling away at the bone with the 10–20 kHz pulse repetition frequency of the regenerative amplifier. Drilling a channel to a typical depth of ~100 µm takes less than a minute. When acquiring images during ablation, the intense white light and SHG signals created by the ablating laser pulses can produce a noise pattern in the image. To remove the noise pattern, the repetition rate of the regenerative amplifier is synchronized to the imaging line scan frequency (17.6 kHz), so that precisely one ablating laser pulse is fired at the end of each scan line and the noise is suppressed during the blanking period (Supplementary Movie 3).

The zone of tissue damage created by the laser ablation procedure can be assessed by examining the integrity of the vasculature near the site of ablation. The BM contains an extremely dense vascular network, which makes up approximately 25–30% of the BM by volume^3, 15, 16^. The blood vessels are easily disrupted if they are directly exposed to the ablation beam^12^. By carefully avoiding direct exposure of blood vessel to the ablating laser beam, the microchannel can be successfully drilled while blood vessels adjacent to or below the ablation site remain intact with unimpeded blood flow (Fig. 2c,d and Supplementary Movie 4).

### Laser-assisted cell delivery to the bone marrow

**Figure 3:**
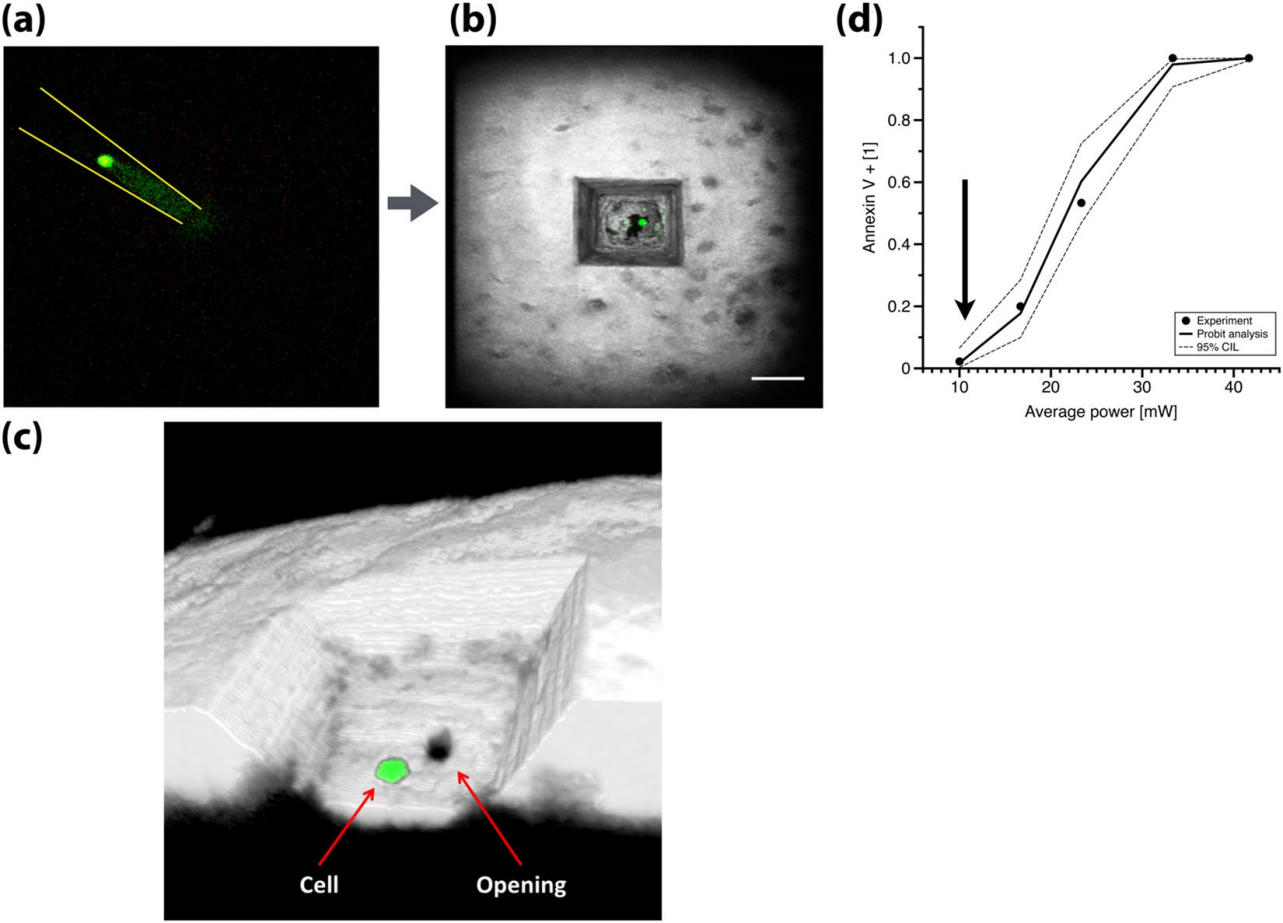
Cell delivery. (**a**) A fluorescence-expressing cell in a glass micropipette is brought on top of the microchannel where it is transferred to the optical tweezer for positioning. At the end of the procedure, a single HSC sits at the bottom of the microchannel: (**b**) top-view, (**c**) 3D reconstruction. (**d**) Assessment of cell death with Annexin-V staining after individual LKS cells were optically trapped for 20 seconds at different powers on a microscope slide (Methods). Arrow points to the maximum power used for cell delivery experiments. Image (**b**) is a maximum intensity projection. White: SHG, Green: GFP. Scale bars 50 μm.

Here we demonstrated the delivery of single cells with HSCs. A single cell is transferred into the optical trap in the immersion medium above the drilled channel with a micropipette that is mounted on a second micro-manipulator (Fig. 3a). The trapped cell is brought into the channel (Supplementary Movie 5) by translating the mouse stage relative to the stationary optical trap. Instantaneous feedback by real-time visualization is crucial for guiding the cell through the narrow delivery channel. At the end of the manipulations, a single cell sits at the bottom of the channel at the intersection with the BM (Fig. 3b,c). Cells have to be delivered within minutes after the opening of the channel because the channel can be blocked by debris.

Viability of optically trapped cells was assessed by the Annexin V assay^17^ (Methods). At the power typically used for delivery, more than 98% of the trapped HSPCs remain viable (Fig. 3d). On the other hand, 100% of the HSPCs died instantaneously at a power 3.5 times larger. This can be taken advantage of when cell delivery fails, i.e. cells occasionally get stuck to the bone wall before they reach the bottom of the microchannel and have to be destroyed to allow a new cell to be delivered (Supplementary Movie 6).

### Longitudinal tracking of single engrafted cells

**Figure 4:**
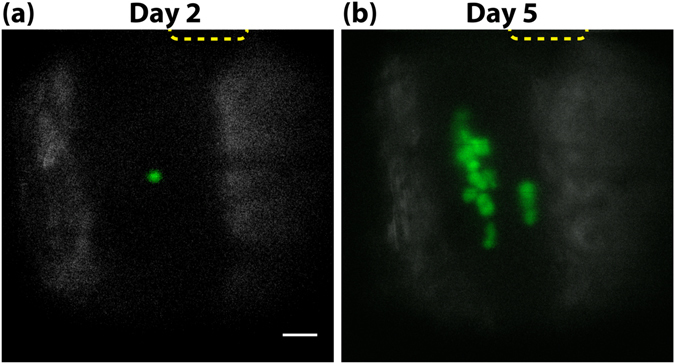
Monitoring local proliferation after single HSPC transplantation. (**a**) Image taken near the delivery site 2 days after transplantation of a single LKS cell into the BM. The image was taken ~100 µm below the bone surface. (**b**) The same location imaged 5 days after the transplantation. To improve image quality the bone was thinned down to ~15 µm. White: SHG, Green: GFP. Scale bar 50 μm. The dotted squares at the top of the images indicate the location of the laser microsurgery, i.e. the delivery site.

In order to fully demonstrate longitudinal tracking of delivered HSCs both the local engraftment and long-term functional outcome need to be observed. To determine whether single delivered cells would penetrate fully into the bone marrow and engraft, intravital microscopy was performed on days 2 and 5 after transplantation. Example of successful single HSPC transplantation into the calvarial BM *in vivo* is shown in Fig. 4a,b and Supplementary Movie 7. A single lineage^low^, c-Kit^+^ and Sca1^+^ (LKS) cell was delivered into a lethally irradiated recipient C57BL/6 mouse and its tracking was possible based solely on the GFP expression. Since GFP is only expressed in the donor and not the host cells, the multiple GFP cells observed at Day 5 provide clear evidence of local proliferation from the single transplanted cell.

**Figure 5:**
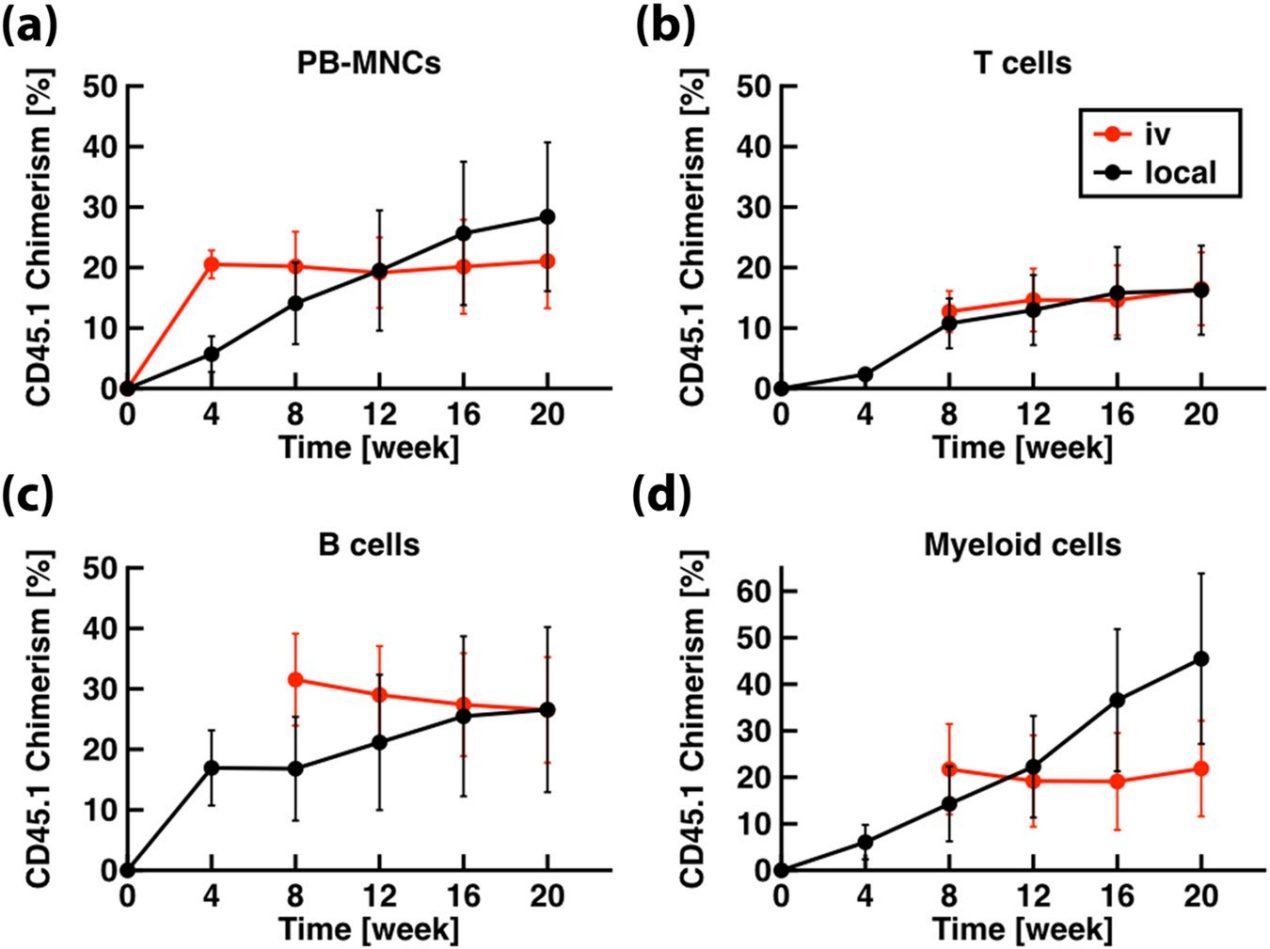
Long-term multi-lineage hematopoietic reconstitution. The dynamics of peripheral blood reconstitution for (**a**) peripheral blood mononuclear cells (PB-MNC), (**b**) T cells, (**c**) B cells, (**d**) and myeloid cells (CD11B^+^) is shown as the average chimerism as a function of time after transplantation of a single Tie2^+^CD150^+^CD48^low/−^CD135^−^ LKS HSC (n = 5, SEM).

To test the long-term engraftment capacity of locally delivered cells, we delivered single HSCs (Tie2^+^CD150^+^CD48^low/−^CD135^−^ LKS) from donor mice expressing the CD45.1 form of the panleukocyte antigen into recipient mice expressing the CD45.2 isoform^18^. In addition, 2 × 10^5^ whole BM cells from CD45.1/CD45.2 donor mice were co-transplant intravenously for short-term support. Since the donor cells had weak GFP expression (driven by the Tie2 promoter), the cells were co-labelled with the membrane dye DiI to facilitate visualization. A continuous-wave laser at 980 nm was also added to the optical platform to serve as a gentler optical trap (Supplementary Fig. 2)^19, 20^. Using this strategy, we obtained long-term and multi-lineage (T cells, B cells, and myeloid cells) hematopoietic reconstitution maintained for at least five months in all local transplantation recipients (Fig. 5, n = 5), with CD45.1 cells contributing 28 ± 12% of peripheral blood cells at week 20, ranging from 1.3 to 62.8%. The chimerism level measured after local transplantation was compared to I.V. transplantation results. The two methods yielded a similar level of peripheral blood chimerism 20 weeks after transplantation (21 ± 8%, n = 9, Wilcoxon rank sum test, p-value = 0.90).

**Figure 6:**
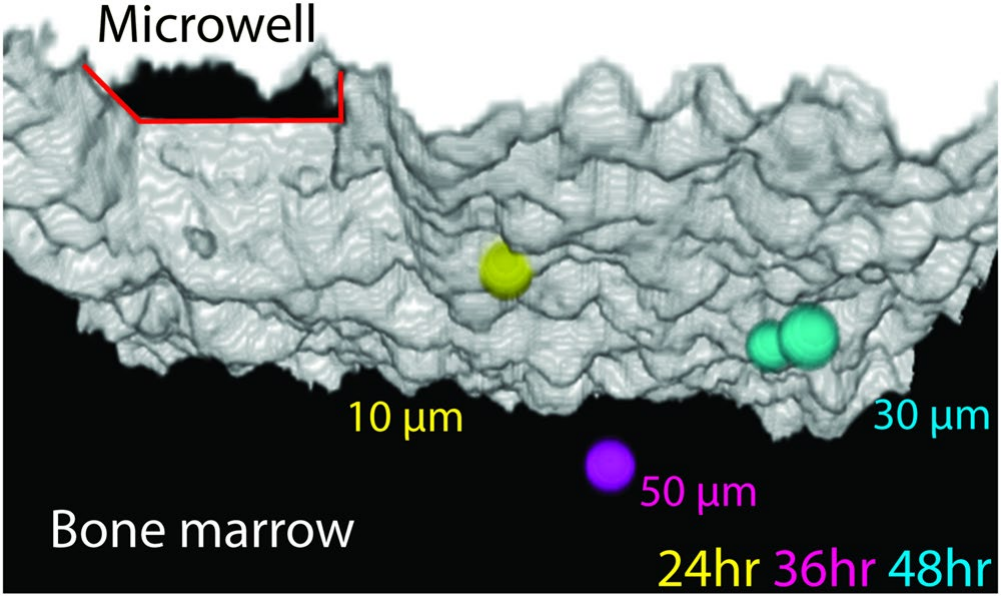
Monitoring engraftment after single HSC transplantation. Three-dimensional reconstruction from intravital imaging of the calvarial BM in the vicinity of the delivery site showing the delivered cell at 24 hours (yellow), 36 hours (magenta), and the outcome of the first HSC division at 48 hours (cyan). At different times, the cell is at a different distance from the endosteal surface (24 hrs: 10 μm, 36 hrs: 50 μm, and 48 hrs: 30 μm).

By revisiting the same location on subsequent days, we were able to perform intravital single HSC tracking (Fig. 6). Single Tie2^+^CD150^+^CD48^low/−^CD135^−^ cells were always found within 100 µm of the delivery site on follow-up imaging. Notably, we observed the outcome of early HSC division and early dynamics. Typical imaging and FACS data from a single mouse are also shown in Supplementary Fig. 3 and Supplementary Fig. 4. We have further performed secondary transplantations of 1 × 10^6^ CD45.1 cells harvested from the whole BM of primary recipients. FACS analysis of the secondary recipient peripheral blood, only two months after transplantation, shows that 51 ± 5% of blood cells are of CD45.1 origin (Supplementary Fig. 5). These results indicate that the single HSC retains self-renewal capacity after local transplantation into the calvarial BM.

## Discussion

The ultimate test of HSC functionality is the observation of donor-derived cells in the peripheral blood circulation after the transplantation of a single cell. The standard approach for single HSC transplantation studies is to inject the cell intravenously. The maximal engraftment success using this approach has been limited to less than 50%^21, 22^ and it is unclear whether this non-absolute engraftment rate informs on HSC properties or is a consequence of technical manipulations. We have designed an optical platform that bypasses the vascular system and directly delivers cells to the BM. With these tools, a single cell can be placed into the BM of live mice with micrometer precision, enabling direct validation of the transplantation outcome. The observation of CD45.1 cells in the peripheral circulation of all recipient mice implies that the HSCs were able to differentiate and generate functional blood cells. The rate of expansion appears to be independent of the delivery route as evidenced by the equivalently high peripheral blood chimerism level after direct (28 ± 12%) or I.V. (21 ± 8%) single cell transplantation. Contribution of a single transplanted cell to hematopoietic recovery after radiation injury can be heavily affected by the presence of HSCs in the co-transplant or by the survival of host HSCs, i.e. single cell transplantation is a competitive assay. The ~30% chimerism observed in these experiments can be further improved by minimizing competition to the locally transplanted single HSC. More importantly, the secondary transplantation results further demonstrate that HSCs preserved their self-renewal capacity after spatially-controlled transplantation.

Another important limitation of the I.V. method is that the single injected HSC cannot be imaged; only its progeny can be visualized after the stem cell has undergone extensive proliferation^9^, leaving open the question of the initial site of HSC engraftment and how the HSC interacts with its niche. Thus there is need for a technique that can track an individual stem cell from its initial engraftment to its long-term regenerative potential. Here, we showed that a spatially-controlled transplantation strategy is uniquely suited to achieve such a feat because the knowledge of the delivery site enables intravital single cell tracking and the visualization of early engraftment dynamics.

Recent advances in femtosecond laser ablation have been critical to help design a microsurgical technique that minimally disrupts surrounding tissue^11–13^. As demonstrated, it is possible to gain direct access to the BM without damaging the local vasculature. Although BM cells and blood vessels located directly under the microchannel opening will be disrupted, there currently exists no other technique that can achieve such a spatially-limited damage zone. The laser tweezer allows for positioning single cells at the entry of a BM cavity, but not deep within it. Delivering a cell deep in the BM would require ablating all cells located above the desired position, thus altering significantly the microenvironment. Also, because of the high density of blood vessels, it would be practically difficult not to disrupt blood vessels. For these reasons, cells are brought as close as possible to the opening with the laser tweezer and their penetration into the bone marrow is mediated by cell motility.

In summary, we have demonstrated the direct transplantation of single HSCs in the bone marrow of live mice. This novel microsurgery was enabled by integrating together multiphoton scanning microscopy, femtosecond laser ablation and optical tweezers onto a single platform. Intravital microscopy can be performed on subsequent days to track single cells. We believe the technique will be used to assess how the type of cell, the delivery site and the interaction with niche components affect the functional output (e.g. long-term reconstitution potential) of a single delivered cell. Demonstrated here mainly with normal hematopoietic stem cells, the platform could also serve in other biological context, in particular with cancer stem cells^23^.

## Methods

### Animals

All animal experiments were performed in compliance with institutional guidelines and approved by the Subcommittee on Research Animal Care (SRAC) at Massachusetts General Hospital. Actin-GFP knock-in mice (C57BL/6-Tg(CAG-EGFP)131Osb/LeySopJ), Actin-DsRed knock-in mice (Cg-Tg(CAG-DsRedMSTNagy/J)), 129Sv-CD45.2 and C57BL/6 mice were purchased from The Jackson Laboratory (Bar Harbor, ME), bred and maintained at the MGH Animal Research Facility. All single-HSPC recipients were 8–10 week-old C57BL/6 mice. HSPC recipient mice received 9.5 Gy of gamma irradiation from a Cs137 source in a single dose 24hrs before single cell delivery. Donor mice were sacrificed by CO_2_ asphyxiation. All single-HSC recipients were 12 week-old 129Sv-CD45.2 mice. HSC recipient mice were irradiated with two doses of 4.5 Gy, delivered 4 hours apart and 24hrs before single cell delivery. HSC donor mice (FVB/129Sv-CD45.1) and co-transplant donor mice (FVB/129Sv-CD45.1/CD45.2) were bred, maintained, and euthanized at the Albert Einstein College of Medicine^18^. The surgical procedure to expose the skull for imaging and local transplantation did not have a significant deleterious effect on survival or long-term health. 90% of mice survived to split-dose irradiation for at least 4 weeks (n = 10, two doses of 450 cGy, 4 hours apart) after retro-orbital intravenous transplantation. When performing local transplantation and imaging, the four-week survival decreased to 63%, but the difference is not statistically significant (n = 8, p-value = 0.16, chi-square test to compare two proportions, two-sided, alpha = 0.05). The laser microsurgery itself doesn’t appear to affect the health status of recipient mice and the microsurgery site is repaired in ~1 month.

### HSPC isolation

Actin-GFP mice were used as donors. The femurs, tibias and hips were dissected out, soft tissue removed, and single-cell suspension was prepared as follows: The bones were crushed, and the marrow cells suspended in ice-cold PBS (Phosphate buffered solution, calcium- and magnesium-free) supplemented with 2% fetal bovine serum (FBS). The cell suspensions were sequentially filtered through 70 and 40-micron cell strainers (BD Falcon) to remove debris. The filtrate was suspended in 50 mL of ice-cold PBS (2% FBS) and then pelleted by centrifugation at 400 g for 10 min. The bone marrow cells were then resuspended in PBS (plus 2% FBS). The lineage negative (Lin^−^) cell population was isolated using MACS® columns (Miltenyi Biotec). Cells were incubated with Lin cocktail (biotinylated antibodies against CD11b, Ter119, CD4, CD8a, CD3e, B220/CD45R, Ly-6G + Ly-6C) (BD Biosciences, San Jose, CA) for 15 minutes. Cells were then washed with PBS-2% FBS. Streptavidin MicroBeads (Miltenyi Biotec) were added, and cell mixture was quickly vortexed and incubated for 15 min at 4 °C. After incubation, the cell mixture was run through MACS® columns, and the lineage-depleted cells were collected in 15 mL tubes.

Fluorescence activated cell sorting of HSPCs was achieved by incubating cells with the antibody specific for Sca-1 (PE-Cy7), c-kit (APC) and Lin (Pacific Orange) for the last 15 minutes (Last four reagents were purchased from BioLegend®). Cell sorting was performed on BD FACSAria (BD biosciences inc) using FACSDiva™ software. HSPC gates were defined as positive for Sca-1, c-kit, and negative for lineage markers and DAPI (Invitrogen/Life Technologies, Carlsbad, CA). HSPCs were automatically collected in 15 mL tubes with 2 mL PBS (2% FBS).

### HSC isolation

HSCs (Tie2^+^CD150^+^CD48^low/−^CD135^−^ LKS) were harvested from the Tie2 GFP mouse and sorted as described in Ito, K *et al*.^18^, using monoclonal antibodies specific for the following: CD41 (eBioMWRag30), CD135 (Avas12a1), CD34 (RAM34), c-Kit (2B8), Sca-1 (E13-161.7), CD3e (145-2C11), CD4 (L3T4), CD8 (53-6.72), B220 (RA3-6B2), TER-119 (TER-119), Gr-1 (RB6-8C5), CD11b (M1/70), IgM (II/41), CD19 (eBio1D3), F4/80 (BM8), CD25 (PC61), CD44 (IM7), CD71 (R17217), CD127 (A7R34), CD45.2 or Ly5.2, (104) CD45.1 or Ly5.1 (A20) and NK-1.1 (PK136); all were from eBioscience. Anti-CD150 (TC15-12F12.2) and CD48 (HM48-1) antibodies were from BioLegend. We used a mixture of monoclonal antibodies against CD4, CD8, CD3e, B220, TER-119, CD11b, Gr-1, IgM, CD19, CD127, and NK-1.1 as a lineage marker (Lineage)^18^.

### Optical delivery platform

The light from a titanium:sapphire laser (80 MHz, Mai Tai, Spectra Physics) running at 800 nm was split 1) for excitation in the multiphoton microscope, 2) to seed a regenerative amplifier (RegA 9000, Coherent) and 3) to provide the optical tweezer (Fig. 1a). Its power was distributed between the three system components using near-infrared (NIR) plate beamsplitters.

1. Multiphoton microscope: The scanning microscope has been described in details elsewhere^10^. Real-time imaging at 30 frames per second was achieved using a spinning polygon and galvanometer mirrors. Three photomultiplier tubes detected the non-descanned signal collected by a 60X 1.0 NA water immersion objective (LUMPLFLN, Olympus). Second-harmonic generation was collected at 400 nm (FF01-417/60-25, Semrock), two-photon excitation fluorescence from GFP at 525 nm (FF01-525/45-25, Semrock) and from rhodamine-B at 600 nm (FF01-607/70-25, Semrock). A 808 nm notch filter (NF03-808E-25, Semrock) eliminated the laser light and dichroics separated multiphoton emission light (720dcxru Chroma, FF458-Di02-25-36 and FF568-Di01-25 × 36, Semrock). A frame grabber card was used to acquire 500 × 500 pixels images. Average power of 25 mW at the sample was used for imaging. In order to position the glass pipette within the field of view of the objective, widefield imaging was performed by illuminating the sample with a white light source located next to the objective, collecting the light leaking through the multiphoton detector arm with a CCD camera.
2. The RegA was seeded by the Mai Tai and pumped by a 10 W, 532 nm continuous wave laser (Verdi, Coherent) and output 400 fs pulses at 10.5 kHz with pulse energy of up to 1.7 µJ. A fluence of 7.5 J/cm^2^ was used to remove tissue up to a depth of 120 µm. Volumetric ablation was achieved by scanning the beam laterally with a 2D galvanometer mirrors set and moving the sample axially. The 2D-scanning unit was synchronized with an automated 3D translational stage via a hardware control card by the image acquisition software. A beam overlap of 1.5 in the fast scanning dimension was used for optimal ablation. A pair of needles (Gauge 26,1.3 cm length, Fisher Scientific) integrated onto the objective lens and connected to a peristaltic pump was circulating the immersion solution (Sodium chloride solution (0.9%)), flushing away ablation debris.
3. The Mai Tai beam used for the optical tweezer was expanded to match the objective back aperture. The optical trap was always remaining stationary and was operated at an average power of 10 mW. Both, the ablation and tweezer beams were coupled into the microscope by a polarizing beam splitter cube (780 nm, 25 mm, Edmund optics) placed just before the objective lens. While the microscope could always be used, the RegA beam and optical tweezer were used alternatively by sliding in and out a set of mirrors placed on a sliding stage. Alternatively, a CW 980 nm laser (L4980M-240-TE/ESYS, Micro Laser System) can be used as the optical tweezers and was introduced onto the laser ablation arm using a bandpass dichroic filter (ZT1064rdc-sp, Chroma) (Supplementary Fig. 2).

### Single cell delivery

Mice were placed in a custom-build heated holder and their head stabilized^24^. The skull was exposed with an incision in the scalp. 50 mL of rhodamine-B,70 kMW dextran (Invitrogen) at 10 mg/mL in sterile sodium chloride solution was injected retro-orbitally to allow vascular imaging. Mice were then brought to the optical delivery platform where SHG from the bone collagen was used to localize BM cavities relative to morphological features. A BM cavity was randomly selected as the delivery site and its position was registered relative to the central vein and the coronal suture^4^. The exact location of the delivery site within the selected cavity was chosen such that impact of the laser ablation process on vasculature would be minimized. The size of the BM cavity opening was ~20 µm. The delivery channel had a pyramidal shape because of the use of a high numerical aperture objective lens. Once opening of the BM cavity was confirmed by the absence of SHG signal and the leakage of some BM content, the ablation beam was blocked and the optical tweezer beam brought into the system.

Simultaneously to laser bone removal, cells for delivery were placed on a glass slide under a widefield microscope (BX51W1, Olympus) and aspirated in a straight glass micropipette (28–32 µm diameter, Origio) attached to a pump (SAS11/2-E, Research Instruments). The micropipette was then brought to the delivery platform, mounted in a second 3D translational stage and positioned using the widefield imaging. The immersion fluid circulation was stopped prior entering the micropipette in the immersion fluid. The cell was then slowly released into the optical tweezer and the animal was immediately moved to direct the cell to the bottom of the channel.

After the cell was delivered, imaging was performed every 5 minutes for up to 15 min to ensure that the cell remains at the delivery site. The mouse was then brought back to the surgical procedure area and left in the mouse holder with the head stabilized. The sodium chloride solution was partially removed. A thin layer was left to prevent the skull from drying. An antibiotic ointment (Original triple antibiotic, Walgreens) was applied on the back of the scalp skin flap to minimize the formation of scarring tissue. The suture attaching the scalp skin flap to the neck was cut and sutures were made to fully close the exposed skull area. Suturing was always performed in the same manner (Supplementary Fig. 6) to minimize skin movement over the delivery site. Some antibiotic ointment was applied along the surgical cut before returning the mouse to its cage where it recovered from anesthesia.

### Confocal/multiphoton imaging

The microscope for follow-up imaging was different from the optical delivery one, but uses the same scanning engines, acquisition software, and objective lens (60 × 1.0 NA water immersion) to yield a field of view of 500 × 500 µm^10^. A single PMT is used to detect the SHG signal from bone generated with the Mai Tai light at 840 nm. The system contains two continuous wave lasers: 491 nm (Dual Calypso, Cobolt AB) and 561 nm (Jive, Cobolt AB). GFP signal (Fig. 4) and autofluorescence signal (Fig. 6) were detected at 528 nm (FF01-528/38-25, Semrock). DiI signal (Fig. 6) and autofluorescence signal (Fig. 4) were recorded at 593 (FF01-593/40-25, Semrock). Autofluorescence signal was used to cleanly delineate delivered GFP or DI-labelled cells from autofluorescent host cells, which have a wide emission spectrum. The fluorescence light was detected by PMTs in a confocal detection configuration. The skull was exposed and closed as for single cell delivery, under anesthesia.

### Optical tweezer cell death assay

Annexin V, Alexa Fluor® 594 Conjugate (4 µL, Invitrogen) was added to 100 µL of a LKS cell suspension at 380,000 cell/mL. A well was made on a glass slide with ring-tape and 20 µL of the cell suspension was placed in the well before adding a coverslip on top. Single cells were trapped for 20 sec with varying power of the optical tweezer. Their locations were recorded and they were imaged for 10 min using the 525 nm and 600 nm TPEF channels. At least 30 cells from different experiments were trapped at every power. Probit statistical analysis was performed with Matlab to determine the fraction of cell apoptosis/death as a function of average power.

## Electronic supplementary material

Supplementary information

Supplementary Video 1

Supplementary Video 2

Supplementary Video 3

Supplementary Video 6

Supplementary Video 4

Supplementary Video 5

Supplementary Video 7
