# Supplementary material for: Image-guided transplantation of single cells in the bone marrow of live animals
Source: Sci Rep. 2017 Jun 20;7:3875. doi: 10.1038/s41598-017-02896-6 (PMC5478633; doi:10.1038/s41598-017-02896-6)
Supplement: Supplementary file 1 — Supplementary information [file 41598_2017_2896_MOESM1_ESM.pdf]

Supporting Material

Spatially-controlled single cell transplantation in the bone marrow of live animals

Raphaël Turcotte, Clemens Alt, Judith M Runnels, Kyoko Ito, Juwell W Wu, Walid Zaher, Luke J Mortensen, Lev Silberstein, Daniel C Côté, Andrew L Kung, Keisuke Ito, and Charles P Lin*

*Corresponding author

Charles P Lin, PhD

Advanced Microscopy Program, Center for Systems Biology and Wellman Center for Photomedicine, Massachusetts General Hospital, Harvard Medical School, Boston, MA 02114.

Tel: 617-724-3957, E-mail: charles_lin@hms.harvard.edu

Supplementary Figure 1. Calvarial bone morphology. Photography of a mouse skull after extraction and removal of all soft tissues. The rectangle shows the location of most of the calvarial BM space where HSPC reside. The central and coronal sutures were used as anatomical references to register the position of cells and delivery sites over the different imaging sessions.

Supplementary Figure 2. Schematic of the optical system with CW optical tweezers (see Methods).

Supplementary Figure 3. FACS analysis of multi-lineage hematopoietic contribution to peripheral blood. Typical FACS data from peripheral blood analysis of a single HSC 12 weeks after local transplantation showing multi-lineage reconstitution in lymphoid (B and T cells) and myeloid lineages ($CD11B^+$ and $Ly6G^+$).

Supplementary Figure 4. Intravital HSC tracking after local transplantation**.** The image shows the single HSC 24 hours after direct transplantation in the calvarial BM from which the data in Supplementary Fig. 3 was derived. Single HSC remained within 100 μm from the delivery site. White: SHG, Red: DiI, and Green: autofluorescence. Image width: 500 μm.

Supplementary Figure 5. Secondary transplantation following spatially-controlled transplantation. Secondary transplantation consisted in the retro-orbital injection of 10^6 CD45.1 cells harvested from the BM of local transplantation recipients. Secondary recipient were irradiated by split dose irradiation the day of transplantation. Each data point for primary recipients represents the peripheral blood chimerism level for a single mouse (week 20). Data points for secondary recipients are the average chimerism for all recipients from a primary recipient (week 11, n=1 to 5).

Supplementary Figure 6. Scalp surgery description. The skull calvarial bone was exposed by making 3 incisions in the scalp (arrows) in order to make a rectangular skin flap. Post-delivery closure of the scalp with minimal skin movement was achieved by making 7 sutures (x) always in the order shown.

Supplementary Video 1. Real-time scanning of the calvarial bone. Second harmonic generation (SHG) signal from the bone allowed to localize BM cavities on the calvarial bone. The central suture is first identified and followed to find the coronal suture. This identification of morphological features allowed to work with anatomically well-defined BM cavities. White: SHG. Field of view 360 μm, imaging speed 30 frame/sec.

Supplementary Video 2. Live laser ablation of a cell delivery channel. Live mouse bone ablation is performed with the animal head stabilized. A flushing system removes all debris from the microsurgery site. Removing debris is a critical step when performing rapid ablation (10.5 kHz). The total ablation time is less than a minute. The dots in the video are due to the strong SHG and white light generation from the ablation laser pulses. Our maximum ablation depth is limited here by the high aspect-ratio of the channel, leaving bone in the light path that cause significant scattering. White: SHG. Field of view 360 μm, imaging speed 30 frame/sec.

Supplementary Video 3. Simultaneous imaging and ablation, free of ablation related noise. The regenerative amplifier pulse frequency can be adjusted between 10 to 250 kHz. The output frequency can be selected such that it corresponds to the line rate of the scanning microscope, in this case 17.6 kHz. This generates a single vertical line of noise originating from the ablation beam that can be synchronized with the frame capture and placed in the blanking period between successive scans (invisible in the recorded image). White: SHG. Field of view 360 μm, imaging speed 30 frame/sec.

Supplementary Video 4. Femtosecond plasma-mediated laser ablation minimally impacts the BM vasculature. The video shows a z-stack of the BM vasculature where the vascular dye was injected 1 hr after ablation. Flow was observed in blood vessels located directly under the ablation site, but not in direct contact with the endosteum. White: SHG, Red: Rhodamine-6G,70kD dextran. Field of view 360 μm, 2 μm axial step between frames.

Supplementary Video 5. Live transfer of a cell to the optical tweezer and cell positioning. The glass micropipette loaded with HSPC GFP+ cell was positioned such that its tip was at the location of the optical tweezers. One cell was slowly released from the micropipette into the optical trap and then guided to the top of the BM cavity by raising the mouse stage. White: SHG, Green: GFP. Field of view 360 μm, imaging speed 30 frame/sec.

Supplementary Video 6. Targeted killing after failed cell delivery. The transfer of a single cell from the glass micropipette to the optical trap can fail. In such cases, cells can fall out of the micropipette and settle onto the bone surface. As the control of the number of delivered cell is critical, miss-delivered cells have to be removed. This is accomplished by illuminating the cell with the optical tweezers beam at maximum power. White: SHG, Green: confocal GFP, Red: autofluorescence – 600 nm. Field of view 360 μm.

Supplementary Video 7. Axial stack of HSPC proliferation 5 days after single cell delivery. White: SHG, Green: confocal GFP. Field of view 500 μm, 2 μm axial step between frames.

Calvarial bone morphology

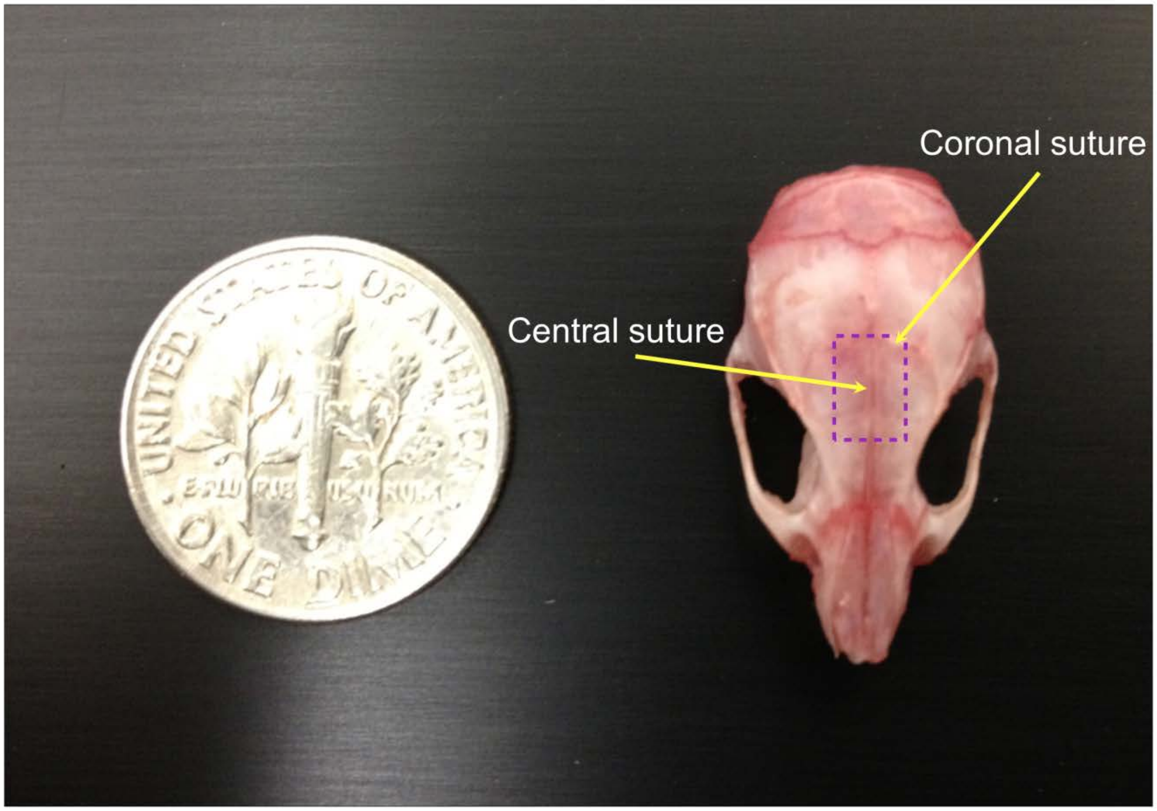

Supplementary Figure 1

Optical system schematic for CW optical tweezers

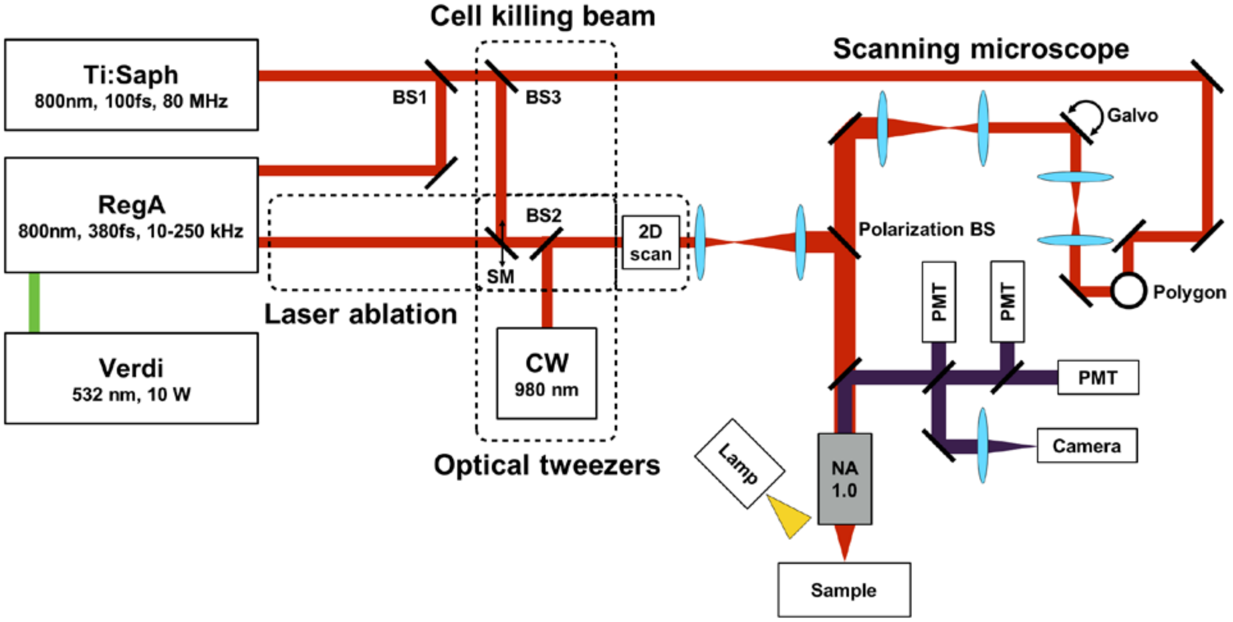

Supplementary Figure 2

FACS analysis of multi-lineage hematopoietic contribution to peripheral blood.

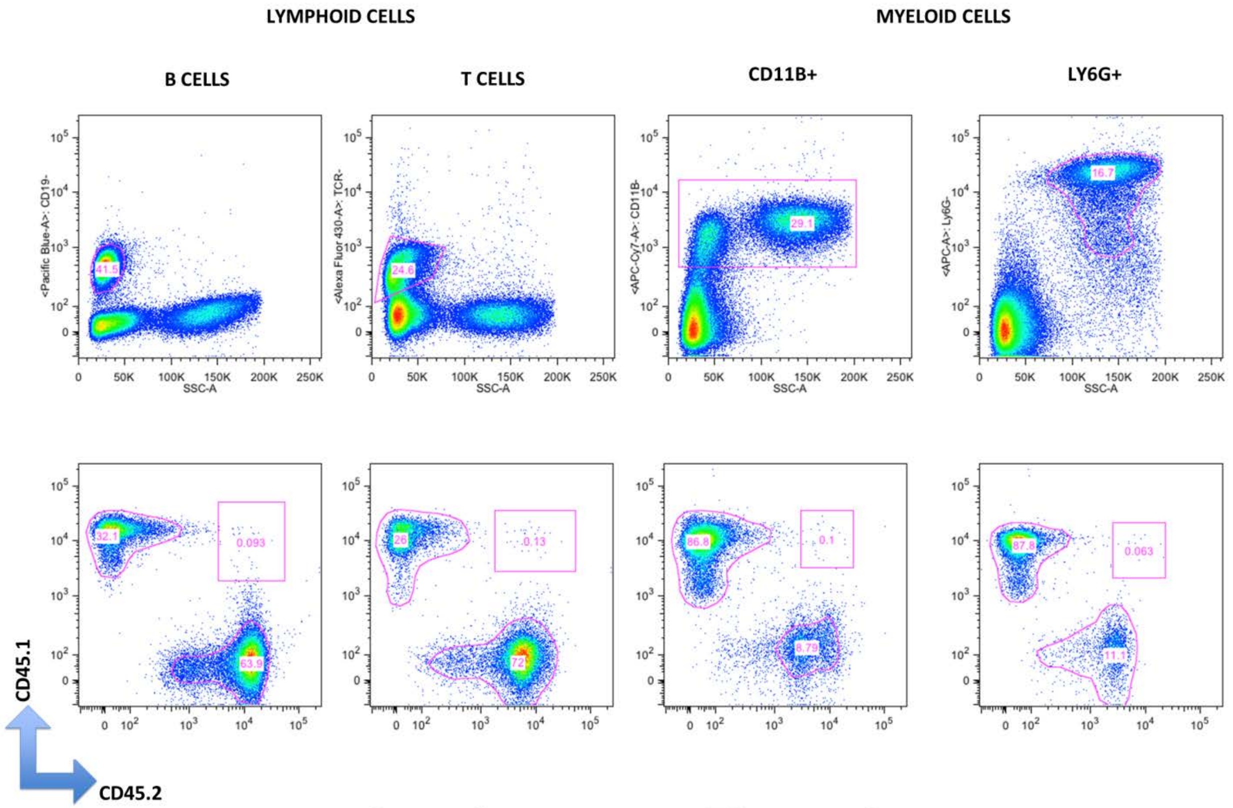

Supplementary Figure 3

Intravital single HSC tracking after local transplantation

24 hours

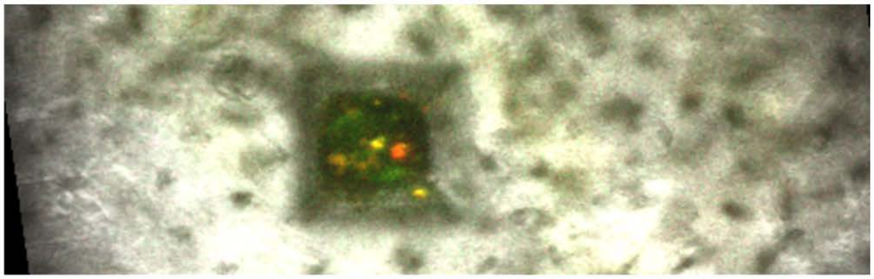

36 hours

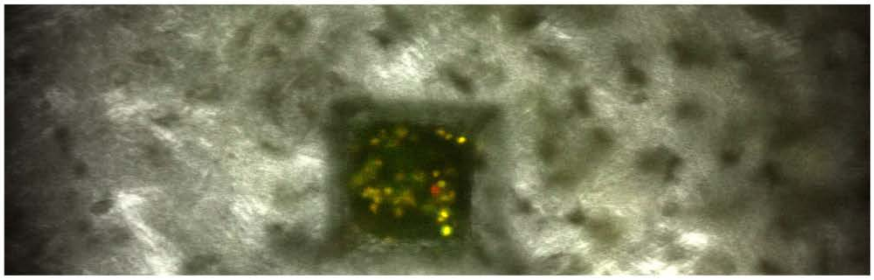

Supplementary Figure 4

Secondary transplantation following spatially-controlled transplantation

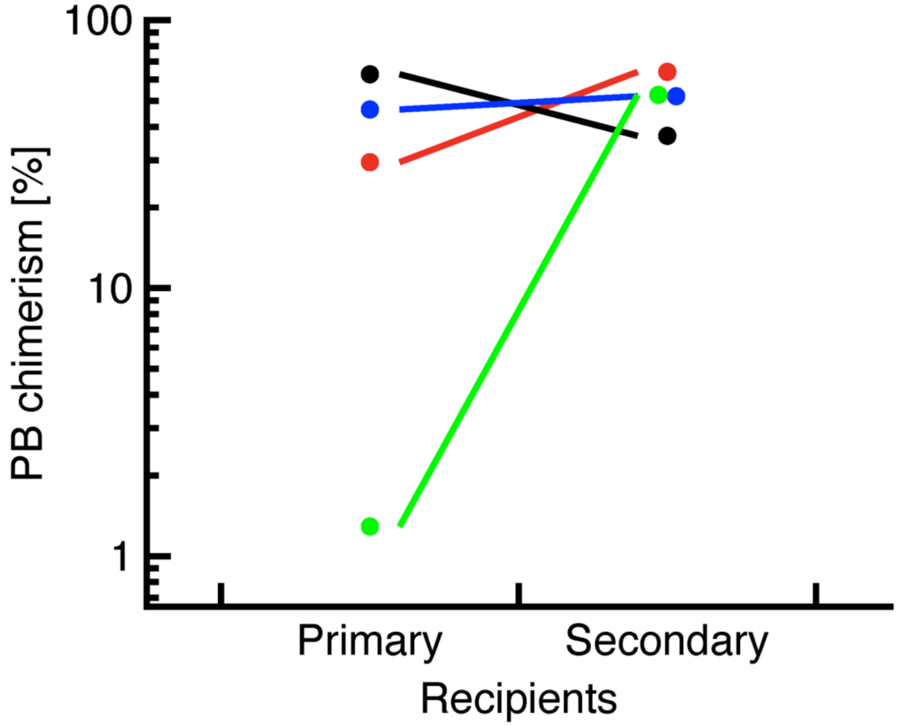

Supplementary Figure 5

Scalp surgery description

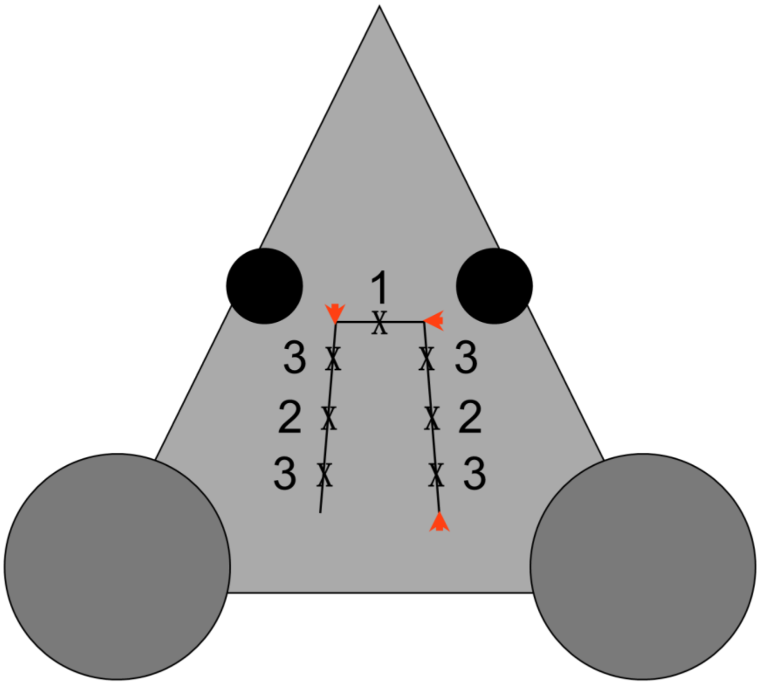

Supplementary Figure 6
